# Epigenetic upregulation of TET2 is an independent poor prognostic factor for intrahepatic cholangiocarcinoma

**DOI:** 10.1007/s00428-021-03251-x

**Published:** 2021-12-14

**Authors:** Hironori Yamashita, Aikaterini Tourna, Masayuki Akita, Tomoo Itoh, Shilpa Chokshi, Tetsuo Ajiki, Takumi Fukumoto, Neil A. Youngson, Yoh Zen

**Affiliations:** 1 Institute of Liver Studies, King’s College Hospital, London, UK; 2 King’s College London, London, UK; 3 Institute of Hepatology, Foundation for Liver Research, London, UK; 4 Department of Hepato-Biliary-Pancreatic Surgery, Kobe University Graduate School of Medicine, Kobe, Japan; 5 Department of Diagnostic Pathology, Kobe University Graduate School of Medicine, Kobe, Japan; 6 Faculty of Life Sciences and Medicine, King’s College London, London, UK; 7 School of Medical Sciences, UNSW Sydney, Sydney, Australia

**Keywords:** Cholangiocarcinoma, TET2, IDH1, Methylation, Promoter

## Abstract

Mutations in *IDH1/2* and the epigenetic silencing of *TET2* occur in leukaemia or glioma in a mutually exclusive manner. Although intrahepatic cholangiocarcinoma (iCCA) may harbour *IDH1/2* mutations, the contribution of TET2 to carcinogenesis remains unknown. In the present study, the expression and promoter methylation of TET2 were investigated in iCCA. The expression of TET2 was assessed in 52 cases of iCCA (small-duct type, *n* = 33; large-duct type, *n* = 19) by quantitative PCR, immunohistochemistry (IHC) and a sequencing-based methylation assay, and its relationships with clinicopathological features and alterations in cancer-related genes (e.g., *KRAS* and *IDH1*) were investigated. In contrast to non-neoplastic bile ducts, which were negative for TET2 on IHC, 42 cases (81%) of iCCA showed the nuclear overexpression of TET2. Based on IHC scores (area × intensity), these cases were classified as TET2-high (*n* = 25) and TET2-low (*n* = 27). The histological type, tumour size, lymph node metastasis and frequency of mutations in cancer-related genes did not significantly differ between the two groups. Overall and recurrence-free survival were significantly worse in patients with TET2-high iCCA than in those with TET2-low iCCA. A multivariate analysis identified the high expression of TET2 as an independent prognostic factor (HR = 2.94; *p* = 0.007). The degree of methylation at two promoter CpG sites was significantly less in TET2-high iCCA than in TET2-low iCCA or non-cancer tissue. In conclusion, in contrast to other IDH-related neoplasms, TET2 overexpression is common in iCCA of both subtypes, and its high expression, potentially induced by promoter hypomethylation, is an independent poor prognostic factor.

## Introduction

The findings of recent pathological studies, including ours, suggested a dichotomous classification scheme for intrahepatic cholangiocarcinoma (iCCA) with distinct clinicopathological features [1, 8, 13]. Small-duct iCCA histologically characterised by cancer cells arranged in an anastomosing tubular architecture is associated with a history of chronic liver disease (~ 50%), a mass-forming appearance on imaging modalities (~ 90%) and a favourable prognosis (5-year overall survival rate, ~ 60%) [1, 2]. In contrast, large-duct iCCA consisting of duct-forming adenocarcinomas with highly fibrotic stroma shows periductal infiltration, frequent lymph node metastasis and a poor prognosis (5-year overall survival rate, ~ 20%) [1, 2]. This separation scheme has been endorsed by the World Health Organization (WHO) classification of tumours published in 2019 [19].

The two iCCA types have several distinct patterns of genetic abnormalities [1, 8, 13]. Unique molecular events in small-duct iCCA include mutations in *IDH1*, *IDH2* and *BAP1* and the fusion or translocation of *FGFR2*, while molecular abnormalities in large-duct iCCA are shared with those in extrahepatic cholangiocarcinoma with commonly observed mutations in *KRAS* and *SMAD4* [1, 8, 13]. We previously detected the amplification of *MDM2* in 15% of large-duct iCCA and perihilar cholangiocarcinoma, but not in small-duct iCCA [9]. However, these unique molecular alterations, which drive tumourigenesis, may be present in up to 50% of iCCA, suggesting that epigenetic alterations are also involved in carcinogenesis. Frequent genetic alterations in epigenetic regulators (e.g., *IDH1*, *IDH2* and *ARID1A*) in iCCA also support the importance of DNA methylation changes in the development and progression of iCCA [21].

Tet methylcytosine mioxygenase 2 (TET2) is an α-ketoglutarate-dependent enzyme that catalyses the conversion of 5-methylcytosine (5mC) to 5-hydroxymethylcytosine (5hmC), thereby regulating gene expression and promoting DNA demethylation [25]. Similar to *IDH1/2* mutations, mutations in and the promoter hypermethylation of *TET2* have been confirmed in leukaemias, myelodysplastic syndrome, myeloproliferative neoplasms and low-grade diffuse gliomas [4, 6, 10]. These alterations in *IDH1/2* and *TET2* are associated with epigenetic defects and a hypermethylation signature [6]. Previous studies reported that an *IDH1/2* mutation was associated with a better prognosis in patients with glioma, acute myeloid leukaemia or iCCA [16, 20, 23, 27, 28].

Therefore, the present study investigated the expression of TET2 and methylation of the *TET2* promoter in iCCA, with the aim of clarifying the clinicopathological significance of molecular alterations in TET2 in iCCA.

## Materials and methods

### Case selection

The present study was approved by the Ethics Committees at our institutes. Fifty-two consecutive patients with iCCA who underwent surgical resection at Kobe University Hospital between 2000 and 2016 were identified in the pathology archives. None had received neoadjuvant chemotherapy prior to surgery. Formalin-fixed paraffin-embedded (FFPE) tissue was used for RNA/DNA extraction and immunohistochemistry (IHC).

Histology slides of surgically resected specimens were reviewed, and cases of iCCA were classified into the small- and large-duct types according to previously described criteria and the WHO classification [1, 19]. Clinicopathological findings were obtained from electronically stored clinical records and histopathology reports. The findings of sequencing of *KRAS* and *IDH1/2* and IHC for BAP1, p53 and SMAD4 in a previous study were used in the present study [1]. Mutations in *KRAS* (exons 2 and 3), *IDH1* (codon 132) and *IDH2* (codon 172) were analysed by Sanger sequencing using DNA extracted from FFPE tissue and following primers: forward 5′–AGGCCTGCTGAAAATGACTG–3′ and reverse 5′–GGTCCTGCACCAGTAATATGCA–3′ for exon 2 of *KRAS*; forward 5′–CCAGACTGTGTTTCTCCCTTCTC–3′ and reverse 5′–AGAAAGCCCTCCCCAGTCCTCA–3′ for exon 3 of *KRAS*; forward 5′–AAACAAATGTGGAAATCACC–3′ and reverse 5′–TGCCAACATGACTTACTTGA–3′ for *IDH1* codon 132; forward 5′–AGAAGATGTGGAAAAGTCCC–3′ and reverse 5′–CAGAGACAAGAGGATGGCTAGG–3′ for *IDH2* codon 172. Post-PCR-amplified products were separated by capillary electrophoresis on an ABI 3130xl Genetic Analyzer (Applied Biosystems, Foster City, CA, USA). Sequencing results were analysed using Sequencing Analysis 5.2 and SeqScape software (Applied Biosystems). IHC for BAP1, p53 and SMAD4 was conducted on one representative whole section in each case. Antibodies used were as follows: BAP1 (clone C–4; dilution 1:100, Santa Cruz Biotechnology), p53 (clone DO–7; dilution 1:300, Leica Microsystems, Wetzlar, Germany) and SMAD4 (clone B–8; dilution 1:100, Santa Cruz Biotechnology, Santa Cruz, CA). The completely negative nuclear staining of BAP1 was defined as a loss of expression, suggesting a BAP1 mutation. Diffuse nuclear staining of p53 was regarded as a positive result, indicating a p53 mutation. Cases with no detectable cytoplasmic or nuclear SMAD4 protein were scored as negative for SMAD4.

### IHC

Tissue microarrays (TMAs) were constructed using 3 tissue cores (2 mm in diameter) obtained from one representative FFPE block of each case. Tissue cores were randomly obtained from tumour masses.

IHC for TET2 was performed using a Bond Max autostainer (Leica Microsystems) according to the manufacturer’s protocol. Deparaffinised sections were subjected to a heat pretreatment in citrate buffer for 20 min, followed by an incubation with a primary antibody against human TET2 (clone N2-2; dilution 1/100; GeneTex, Irvine, CA, USA). TET2 expression was observed in the cytoplasm and nuclei. Given that TET2 is a nuclear protein, only nuclear staining was assessed. Nuclear expression levels in TMA were semiquantitatively evaluated based on the percentage of positive cancer cells (score 0 = 0%; score 1 = 1–33%; score 2 = 34–66%; score 3 = 67–100%) and the intensity of expression (score 0 = negative; score 1 = weak; score 2 = moderate; score 3 = strong). Weak intensity of the expression (score 1) was defined as faint staining requiring examination at a high-power magnification (× 40 objective lens), while strong intensity of the expression was defined as brisk staining with clear contrast to the background. Moderate intensity (score 2) was defined as staining between scores 1 and 3. IHC scores in individual cases were assessed by multiplying percentage and intensity scores (range 0–9). In cases, in which TET2 was negative in TMA, additional staining on whole sections was performed.

### Reverse-transcription quantitative PCR (RT-qPCR)

Areas consisting predominantly of tumour cells were selected for RNA extraction under a microscope. The background liver parenchyma and large bile ducts (5 cases each) were used as a control group. Total RNA was extracted from FFPE sections using the AllPrep DNA/RNA FFPE Kit (Qiagen, Hilden, Germany). One microgram of RNA was treated with DNAse I Amplification Grade (AMPD1-1KT; Sigma-Aldrich, St. Louise, MO, USA) and then reverse transcribed using a High Capacity cDNA reverse transcription kit (4,368,814, Applied Biosystems) as per the manufacturer’s protocol. Real-time PCR was conducted in triplicate using Luna® Universal qPCR Master Mix (New England BioLabs, Ipswich, MA, USA) as the SybrGreen Probe on an Ariamx Real-Time PCR System (Agilent, Santa Clara, CA, USA). Primer sequences for PCR were as follows: TET2 F GCTTCCATTCTGGAGCTTTG, TET2 R GGACATGATCCAGGAAGAGC. Large group-specific differences were observed in housekeeper genes therefore data presented as sample TET2 Ct from equivalent original input RNA (lower Ct indicates a higher expression level).

### Methylation analysis of the TET2 promoter

Genomic DNA was extracted from areas in FFPE tissue sections that predominantly of tumour cells. DNA was extracted using the AllPrep DNA/RNA FFPE Kit (Qiagen). DNA content was assessed using the NanoDrop 2000c spectrophotometer (Thermo Fisher Scientific, Waltham, MA). Enzymatic conversion was performed on 1 µg of DNA with the Enzymatic Conversion Module (New England Biolabs). Primer sequences for converted DNA were designed by Methprimer [12] and manufactured by Eurofins Genomics (Ebersberg, Germany). Semi-nested PCR was performed with the second primer (inner) containing 5′-prime octamer tags for the sequencing of barcodes. Primer sequences for amplification from enzymatically converted DNA were as follows: TET2 Outer F GGAAGTAAGATGGTTGTTTTTTAGG, TET2 Outer R AAACTTCCCTCTTCCCTCTTAATATT, TET2 Inner F GGAAGTAAGATGGTTGTTTTTTAGG and TET2 Inner R ACACTACAAAATTTACTCCCCAATC. PCR was performed according to the Hot Start protocol for EpiMark Taq polymerase (New England Biolabs). The next-generation sequencing of PCR products was conducted by Amplicon-EZ (Genewiz, Leipzig, Germany), and the data obtained were initially analysed in the Unix shell to separate patients according to octamer barcodes and convert fastq files to fasta, followed by the BiQ Analyzer HT software [15], which revealed the methylation state of each individual CpG site.

### Statistical analysis

Statistical analyses were performed using JMP statistical software (version 12; SAS Institute, Cary, NC, USA). Continuous variables not showing a bell-shaped distribution were assessed using the unpaired *t* test or Mann–Whitney *U* test, whereas categorical variables in each group were compared using the *X*^2^ test or Fisher’s exact test. Survival curves were constructed using the Kaplan–Meier method and compared between two groups by the Log-rank test. Univariate and multivariate analyses of multiple prognostic factors were performed on prognostic factors assessed by the Cox proportional hazards model. In RT-qPCR and DNA methylation analyses, values more or less than 2 standard deviations from the mean were considered to be outliers and, thus, were excluded.

## Results

### Clinicopathological features

Among the 52 cases examined, 33 (63%) were classified as the small-duct type and 19 (37%) as the large-duct type. Eighteen patients (35%) had a history of chronic liver disease and two (4%) had features of established cirrhosis. Tumours were larger than 5 cm in 25 cases (48%) and lymph node metastasis was confirmed in 14 cases (27%). Intrahepatic metastasis including satellite nodules was observed in 16 cases (31%). Molecular or IHC studies confirmed *KRAS* mutations in 9 cases (17%), *IDH1* mutations in 4 (8%), the loss of BAP1 expression in 12 (13%), the diffuse expression of p53 in 13 (25%) and the loss of SMAD4 expression in 7 (13%). *IDH2* was the wild type in all cases.

### TET2 mRNA expression and promoter methylation

**Fig. 1:**
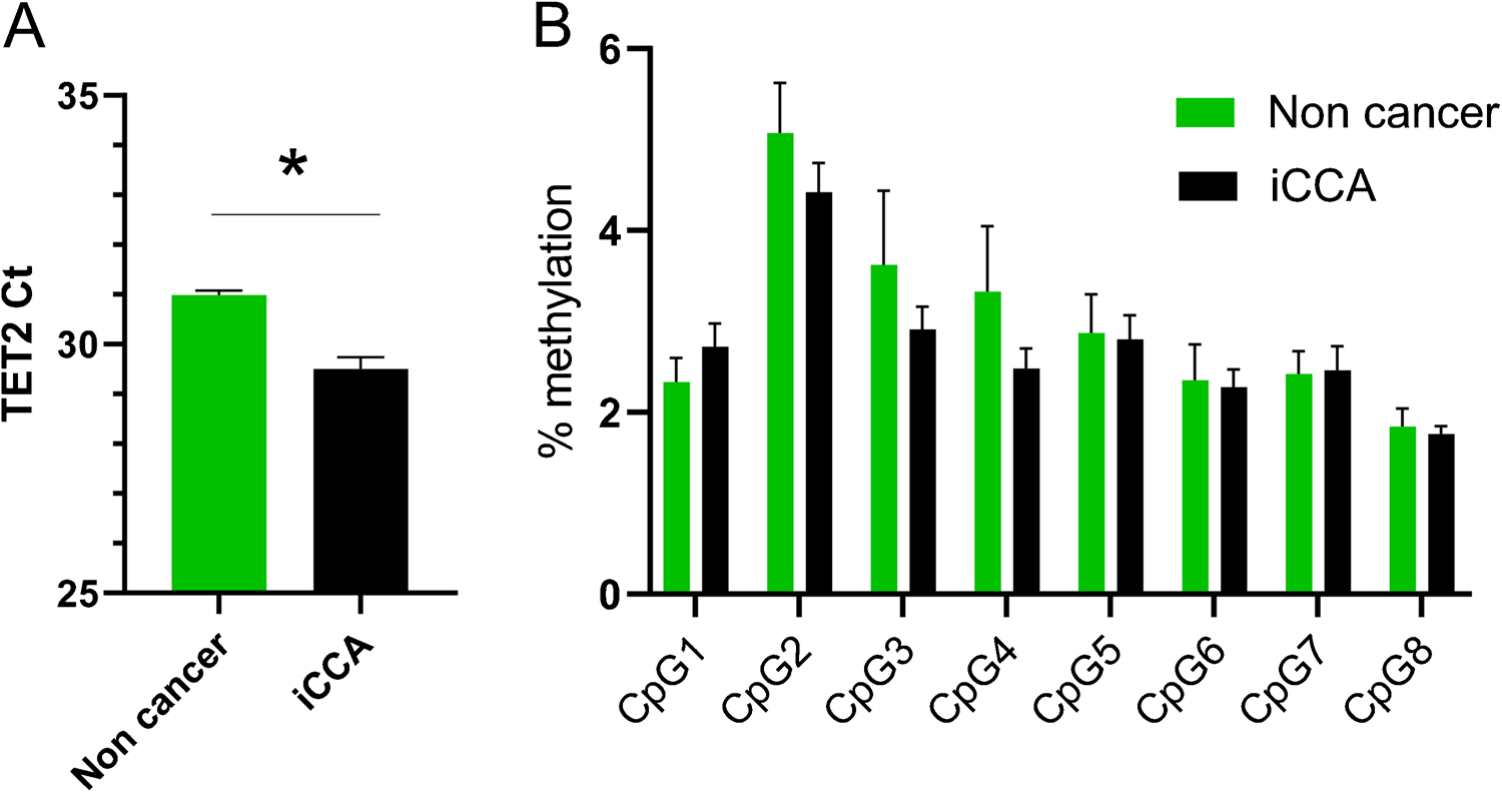
**A** RT-qPCR of TET2. Ct from an equivalent amount of input RNA. Lower Ct means higher expression. Data are the mean ± SEM. *p < 0.05. **B** Methylation levels at 8 CpG sites in the promoter CpG island of TET2. Data are the mean ± SEM. No significant differences at any CpG site

RT-qPCR revealed that the mRNA expression levels of TET2 were significantly higher in iCCA tissue than in background liver/bile duct tissue with a group average Ct difference of 1.5 PCR cycles, equivalent to a 2.8-fold difference (*p* = 0.004; Fig. 1A). However, no significant difference was observed in CpG methylation at 8 CpG sites in the promoter of TET2 when all iCCA samples were compared to non-cancer tissue (Fig. 1B).

### IHC for TET2

**Fig. 2:**
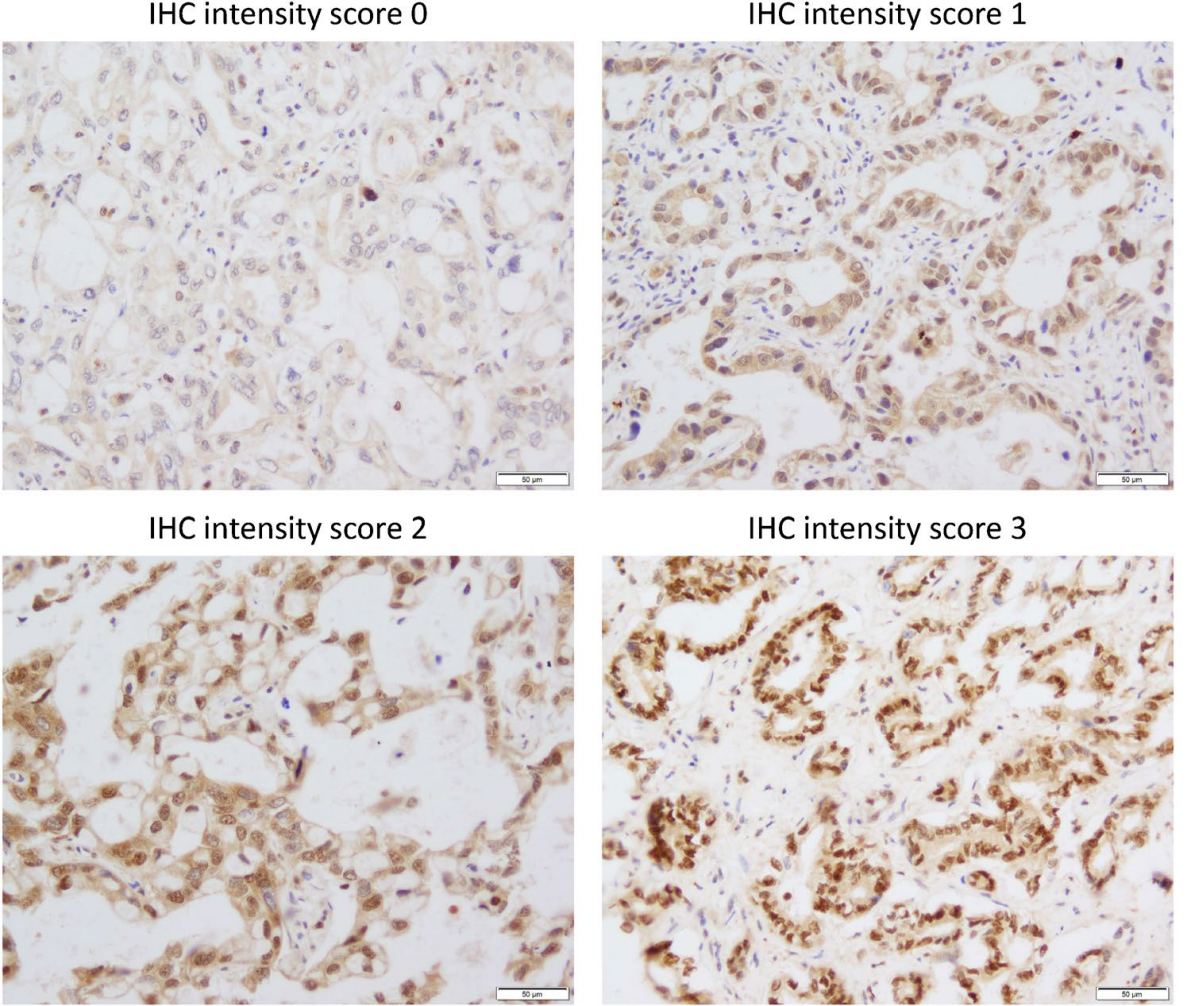
Immunohistochemical expression of TET2 in iCCA. Representative examples of IHC intensity scores 0–3 are shown. Variable degrees of nuclear TET2 expression are observed, and immunoreactivity is also present in the cytoplasm

Immunostaining for TET2 was conducted to examine the protein expression of TET2 in 52 cases of iCCA relative to background liver/bile duct tissue. The non-neoplastic cholangiocytes of the small and large bile ducts did not show nuclear immunoreactivity for TET2. In contrast, 42 cases of iCCA (81%) showed variable degrees of expression in TMA (Fig. 2). TET2 expression was located in the nuclei and cytoplasm, and the nuclear expression was slightly more pronounced than cytoplasmic staining in most cases. The remaining 10 cases of iCCA (19%) were negative for TET2 in TMA. However, additional immunostaining on whole sections of those cases demonstrated focal TET2 expression. Based on IHC scores of TMA, cases were classified as TET2-high (IHC scores 4–9; *n* = 25) and TET2-low (IHC scores 0–3; *n* = 27).

### Comparisons between TET2-high and -low cases

**Fig. 3:**
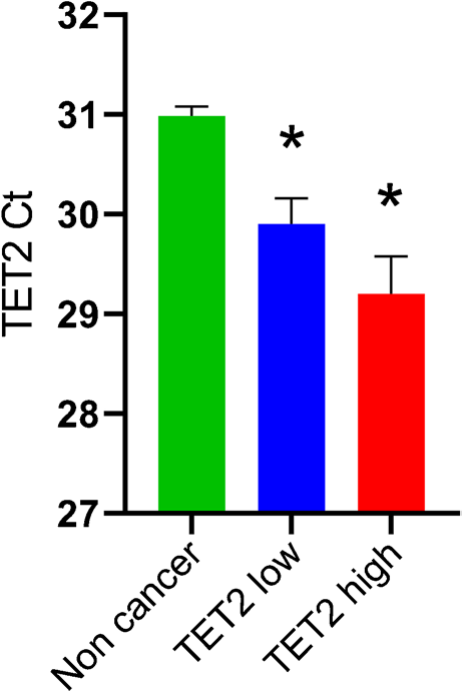
RT-qPCR of TET2. Ct from an equivalent amount of input RNA. Lower Ct means higher expression. Data are the mean ± SEM. *p < 0.05 vs. the non-cancer group

Table 1 compares clinicopathological features between TET2-high and -low cases. No significant differences were observed in any of the parameters examined. No correlations were found between TET2 expression and histological subtypes. The frequencies of *KRAS* or *IDH1* mutations and the loss of BAP1 or SMAD4 expression were also similar. Ct values in TET2 mRNA RT-qPCR on TET2-high cases were not significantly different from those for TET2-low cases (Table 1); however, both were significantly lower than those for non-cancer tissue (Fig. 3).

**Table 1:** Comparison of clinicopathological features between TET2-high- and low cases

	TET2-high	TET2-low	
	(*n* = 25)	(*n* = 27)	*p* value
Age (median, range)	71 (40–86)	70 (43–83)	0.582
Gender (M/F)	14/11	15/12	0.974
Chronic liver disease	7 (28%)	11 (41%)	0.335
CA19-9 (median, range)	157 (1–43,400)	107 (1–829,298)	0.614
CEA (median, range)	3.1 (0.3–27.6)	4.0 (1.0–405.6)	0.384
Subtype			0.282
Small duct	14 (56%)	19 (70%)	
Large duct	11 (44%)	8 (30%)	
Differentiation			0.401
Well to moderate	19 (76%)	23 (85%)	
Poor	6 (24%)	4 (15%)	
Tumour size (> 2 cm)	23 (92%)	23 (85%)	0.442
Tumour size (> 5 cm)	11 (44%)	13 (48%)	0.764
Lymphatic invasion	10 (40%)	12 (44%)	0.746
Venous invasion	19 (76%)	18 (67%)	0.458
pT stage			0.631
pT1-2	18 (72%)	21 (78%)	
pT3-4	7 (28%)	6 (22%)	
Lymph node metastasis	8 (32%)	6 (22%)	0.427
Intrahepatic metastasis	7 (28%)	9 (33%)	0.677
*KRAS*, mutation	4 (16%)	5 (19%)	0.810
*IDH1*, mutation	2 (8%)	2 (7%)	0.936
BAP1, loss of expression	4 (16%)	8 (30%)	0.244
p53, strong expression	8 (32%)	5 (19%)	0.262
SMAD4, loss of expression	4 (16%)	3 (11%)	0.606
TET2, mRNA expression (Ct)	29.2 ± 0.38	29.9 ± 0.26	0.127
*TET2*, promoter methylation	2.31 ± 0.15	2.62 ± 0.21	0.238

### TET2 promoter methylation analysis

**Fig. 4:**
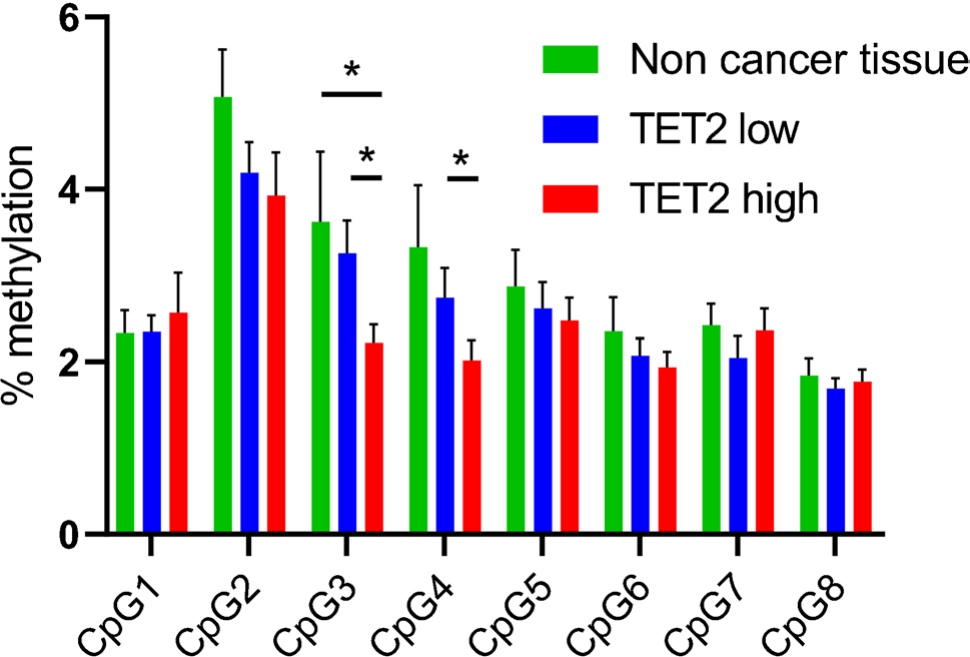
Methylation levels at 8 CpG sites in the promoter CpG island of TET2. Data are the mean ± SEM. Separate *t* tests were used to compare groups at each CpG site. *p < 0.05

In order to examine the relationship between TET2 expression and *TET2* promoter methylation, the methylation of 8 CpG sites in the promoter region was quantitatively assessed. The mean value of methylation in the 8 CpG sites did not significantly differ between TET2-high and -low cases (2.31 ± 0.15% vs. 2.62 ± 0.21%; *p* = 0.238; Table 1). However, a comparison of individual CpG sites revealed that methylation levels at two CpG sites in TET2-high cases were significantly lower than those in TET2-low cases (Fig. 4).

### Prognostic analysis

**Fig. 5:**
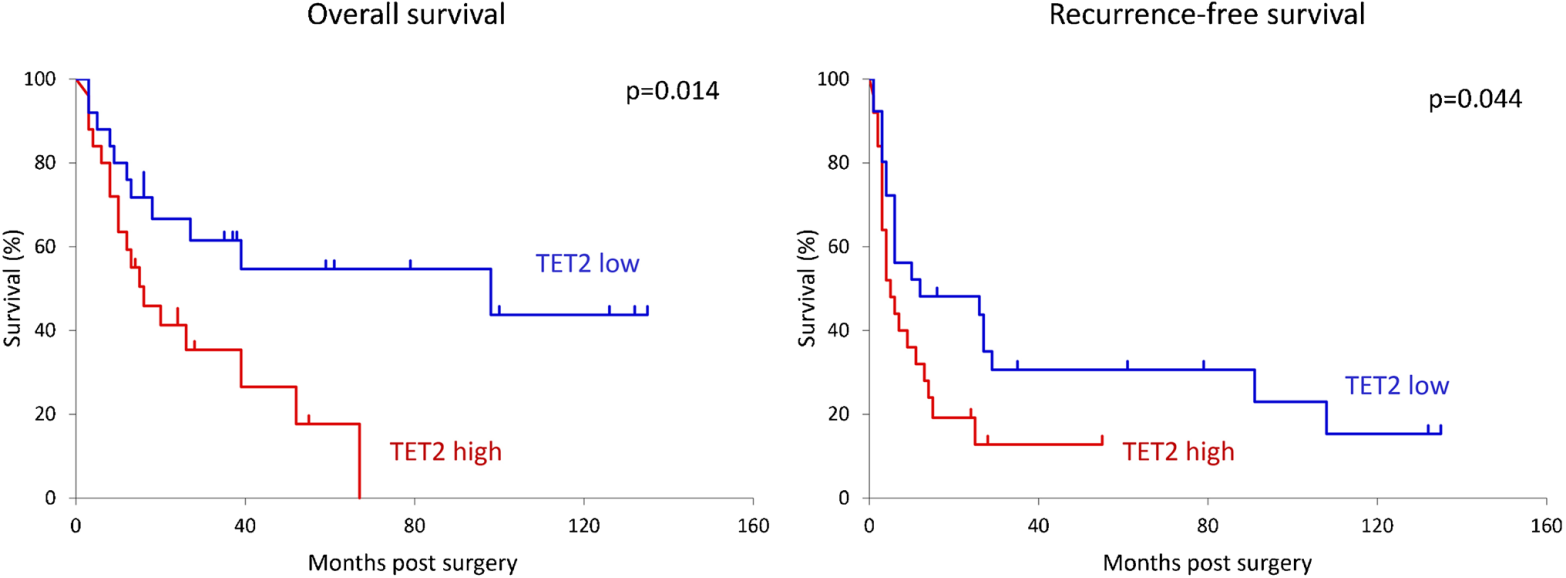
Overall and recurrence-free survival curves of patients with iCCA. Overall and recurrence-free survival were significantly worse in patients with TET2-high iCCA

As shown in Fig. 5, overall and recurrence-free survival were significantly worse in patients with TET2-high iCCA than in those with TET2-low iCCA (*p* = 0.014 or *p* = 0.044, respectively). In a multivariate analysis, TET2 and other potential prognostic factors that showed a significant (*p* < 0.05) or slight difference (*p* < 0.20) in the univariate analysis were applied to the Cox proportional hazards model. TET2 overexpression, the large-duct histological type and intrahepatic metastasis were identified as independent poor prognostic factors in patients with iCCA (Table 2).

**Table 2:** Univariate and multivariate prognostic analysis

	Univariate analysis			Multivariate analysis		
	HR	95% CI	*p* value	HR	95% CI	*p* value
TET2-high expression	2.58	1.19–5.89	0.016	2.94	1.33–6.85	0.007
Large-duct histological subtype	1.91	0.90–4.06	0.092	2.75	1.11–7.18	0.028
Poor differentiated morphology	1.65	0.60–3.90	0.306			
Tumour size > 5 cm	1.77	0.84–3.73	0.129	1.46	0.63–3.46	0.379
pT3 or pT4	1.12	0.44–2.53	0.796			
Lymph node metastasis	1.96	0.84–4.23	0.115	2.20	0.86–5.31	0.096
Intrahepatic metastasis	1.77	0.80–3.71	0.152	3.47	1.28–9.85	0.015

*HR*, hazard ratio; *CI*, confidence interval

## Discussion

The present study demonstrated that approximately 80% of iCCA cases expressed TET2. When these cases were classified into two groups based on IHC scores, the overexpression of TET2 did not correlate with histological types, a history of chronic liver disease, or lymph node or intrahepatic metastasis; however, it correlated with poor overall and recurrence-free survival. A multivariate analysis also identified TET2 overexpression as a prognostic factor independent of the histological type, tumour size, nodal involvement and intrahepatic metastasis. A quantitative methylation analysis of the promoter region suggested that the hypomethylation of two particular CpG sites contributed to the upregulation of TET2 expression in iCCA. The small difference in methylation values between the two groups is expected as the methylation change will only be occurring in the subpopulation of cells in the cancer tissue sample which express TET2. The tissues also contain non-TET2 expressing non-cancer cells (e.g., lymphocytes, stromal cells).

Distinct carcinogenetic processes between small- and large-duct iCCA have been highlighted by histology-molecular correlation studies [1, 8, 13]. Alterations in *IDH1*, *IDH2* and *BAP1* are restricted to small-duct iCCA, while changes in *KRAS*, *SMAD4*, and *MDM2* are more commonly observed in large-duct cancers [1, 9, 13]. However, molecular abnormalities that occur in either type have also been reported. The *TP53* loss-of-function mutation is one of the most common molecular events potentially occurring in either subtype [1, 18]. The epigenetic upregulation of TET2 is also suspected to contribute to the progression of both types of iCCA.

Multiple deep sequencing studies identified recurrent mutations in genes involved in chromatin remodelling (e.g., *IDH1/2*, *ARID1A*, *SMARCA* and *KDMA5A*), highlighting a strong epigenetic component in iCCA carcinogenesis [7, 18]. A combined genetic and epigenetic analysis of iCCA revealed that cases with high mutation and hypermethylation levels had a worse prognosis than those with fewer genetic alterations and lower hypermethylation levels [7]. Epigenetic alterations in multiple genes, including *ROBO1*, *ROBO2*, *RPL22*, *TGFBR1* and *TGFBR2*, have been confirmed in iCCA; however, this is the first study to investigate epigenetic alterations in TET2 in iCCA [7].

Prior to the initiation of the present study, we hypothesised that TET2 may be down-regulated in iCCA, particularly small-duct type cases with wild-type *IDH1*, similar to haematolymphoid malignancies and diffuse glioma [25]. The downregulation of TET2 may exert a similar epigenomic effect as the *IDH1* mutation, namely, an increase in DNA methylation levels. However, we observed the opposite effect; TET2 transcription levels in iCCA were higher than those in background liver/bile duct tissue. The protein expression levels of TET2 also did not correlate with histological types or *IDH1* mutations. These unexpected results may be attributed to the different roles of TET2 in haematopoietic cells and cholangiocytes. TET2 is highly expressed in non-neoplastic haematopoietic cells [11, 14]; therefore, TET2 silencing by promoter methylation disturbs the expression of various genes, ultimately leading to tumourigenesis. In contrast, TET2 expression is suppressed in non-neoplastic cholangiocytes, and, thus, the upregulation of TET2 by promoter hypomethylation may contribute to the development or progression of iCCA.

Increases and decreases in DNA methylation (5mC) and 5-hmC at genes and intergenic regions have both been reported in cholangiocarcinoma [22]. The genome-wide level of 5hmC was previously reported to be decreased in iCCA (rather than increased, as was expected from the upregulated expression of TET2) [5]. However, it currently remains unclear whether these studies examined iCCA highly expressing TET2. The mechanisms by which the overexpression of TET2 influences tumour progression and patient survival may involve the altered regulation of a subset of downstream genes rather than an overall shift in the genome-wide epigenetic state. A focus for future research will be to identify which genes have altered 5mC and/or 5hmC in tumours overexpressing TET2.

A highly relevant study showed increases in the expression of TET2 and 5hmC in a number of cancers that modulated the expression of TNF-α signalling components and facilitated chemotherapy resistance in slow-cycling cancer cells by restraining proapoptotic signalling [24]. This effect of TET2 on a subpopulation of tumour cells with cancer initiation potential, which is not necessarily a factor influencing tumour size or stage, was consistent with our observations that the overexpression of TET2 is a prognostic factor independent of these tumour parameters. Future studies that investigate the effectiveness of TET2 inhibitors, combined with chemotherapeutics against proliferative cancer cells, may be relevant for iCCA research.

Due the high level of *IDH1* mutations, which increase 5mC, a number of studies have attempted to reduce methylation in cholangiocarcinoma using inhibitors in cell lines [3, 17, 26]. The present study raises a note of caution with this approach because it may activate *TET2* by demethylating its promoter and, ultimately, decrease patient survival.

In conclusion, the present study demonstrated that the overexpression of TET2 is common in iCCA of both subtypes, and identified the high expression of TET2 as an independent poor prognostic factor. A quantitative methylation analysis suggested that hypomethylation in two particular CpG sites underlies the upregulation of TET2 in malignant cholangiocytes.

## Abbreviations

5hmC: 5-Hydroxymethylcytosine
5mC: 5-Methylcytosine
CpG: Cytosine guanine dinucleotide
iCCA: Intrahepatic cholangiocarcinoma
IHC: Immunohistochemistry
FFPE: Formalin-fixed paraffin-embedded
WHO: World Health Organization
